# Genome-wide characterization of the *RCI2* gene family reveals conserved protein features and divergent responses to salt and osmotic stress in pea

**DOI:** 10.3389/fpls.2026.2003888

**Published:** 2026-10-29

**Authors:** Mingyuan Zhang, Yongdong Dai, Rui Yang, Hongjuan Wang

**Affiliations:** 1 Biotechnology Research Institute, Chongqing Academy of Agricultural Sciences, Chongqing, China; 2 Tianjin Customs Animal, Plant and Food Inspection Center, Tianjin, China

**Keywords:** osmotic stress, *Pisum sativum*, RCI2 gene family, salt stress, subcellular localization

## Abstract

**Introduction:**

Salinity and drought are major abiotic stresses limiting pea (*Pisum sativum L.*) growth and productivity. The *RCI2* family has been implicated in plant responses to environmental stress, but its characteristics and potential roles in pea remain insufficiently understood.

**Methods:**

Six *PsRCI2* genes were identified and characterized using phylogenetic, gene structure, promoter, transcriptomic, quantitative real-time PCR (qRT-PCR), and subcellular localization analyses.

**Results:**

*PsRCI2-2* to *PsRCI2-5* formed a gene cluster on chromosome 3, whereas no clear intragenomic collinear pairs were detected. Most PsRCI2 proteins showed conserved sequence and transmembrane features, whereas PsRCI2-6 exhibited distinct structural characteristics. Promoter analysis revealed diverse stress- and hormone-responsive cis-acting elements among family members. Transcriptome analysis revealed differential transcriptional responses to salt and PEG-induced osmotic stress. Notably, *PsRCI2-2*, *PsRCI2-3*, and *PsRCI2-5* were induced by both treatments, and their expression patterns were validated by qRT-PCR. These three proteins also exhibited fluorescence patterns largely overlapping with the ER marker in *Nicotiana benthamiana*.

**Discussion:**

The pea *RCI2* family displays conserved structural features but divergent transcriptional responses. *PsRCI2-2*, *PsRCI2-3*, and *PsRCI2-5* are promising candidates for further functional studies investigating pea responses to abiotic stress.

## 1 Introduction

Climate change and soil degradation pose major threats to global food security, while salinity and drought are among the most important abiotic stresses limiting crop growth, development, and productivity (Mukhopadhyay et al., 2021; Tang et al., 2024). Driven by global warming, altered precipitation patterns, unsustainable irrigation practices, and increasingly severe secondary soil salinization, the areas affected by salinity and drought continue to expand, posing serious threats to agricultural production and ecosystem stability. Both salt and drought stresses can disrupt cellular water status and osmotic homeostasis, leading to ionic imbalance, excessive accumulation of reactive oxygen species (ROS), and metabolic dysfunction, which ultimately impair seed germination, root growth, biomass accumulation, and yield formation (Khalid et al., 2023; Whipple et al., 2019). Pea (*Pisum sativum* L.) is an important food and economic crop worldwide, valued for its high protein content and important contribution to biological nitrogen fixation. It plays an important role in global food security and sustainable agricultural development (Palmero et al., 2022; Zhang et al., 2025b). However, pea is highly sensitive to both salinity and water deficit. Salinity can cause ionic toxicity and osmotic imbalance, whereas drought can induce cellular dehydration, reduced photosynthetic capacity, and oxidative damage, ultimately suppressing plant growth and development (Alharbi et al., 2022). Therefore, elucidating the molecular mechanisms underlying abiotic stress tolerance in pea and identifying key regulatory genes are essential for the genetic improvement and breeding of salt-tolerant pea cultivars.

At the cellular level, these stresses disrupt water and ion homeostasis and impose substantial challenges to membrane integrity, highlighting the importance of membrane-associated proteins in plant stress adaptation (Banik and Dutta, 2023; Mani et al., 2025). In this context, the Rare Cold Inducible 2 (RCI2) gene family, also known as Low Temperature Induced 6 (LTI6) or Plasma Membrane Proteolipid 3 (PMP3), encodes a group of highly conserved, small hydrophobic membrane proteins. Most plant RCI2 proteins contain a conserved PMP3 domain and two predicted transmembrane domains and are approximately 54–78 amino acids in length. Some members also possess a hydrophilic C-terminal extension, indicating structural diversity within this otherwise conserved family (Rocha, 2016). The reported sizes of the *RCI2* gene family vary among plant species. Eight *AtRCI2* genes (*AtRCI2A–H*) have been identified in *Arabidopsis*, while 12 non-redundant *OsRCI2* members were reported in rice and 10 *ZmRCI2* genes were identified in maize (Medina et al., 2007; Zhao et al., 2014). In alfalfa, a recent genome-wide study identified six distinct *MsRCI2* loci, with 22 sequences reported when putative allelic variants were included (Qiao et al., 2025).

Functionally, the yeast PMP3 protein was shown to contribute to ion homeostasis by modulating plasma membrane potential, thereby enhancing cellular adaptation to salt stress (Navarre and Goffeau, 2000). Subsequent studies showed that RCI2 proteins are widely distributed across the plant kingdom. They are mainly localized to the plasma membrane and are involved in maintaining membrane stability, membrane potential, and ionic homeostasis (Rocha, 2016). Accumulating evidence indicates that *RCI2* family members are involved in plant responses to salt and drought stresses. In *Arabidopsis thaliana*, *RCI2A* mutants exhibit increased sensitivity to salt and cold stresses, indicating that *RCI2A* plays an important role in plant adaptation to adverse environmental conditions (Medina et al., 2005). Moreover, heterologous overexpression of maize *ZmPMP3–1* and alfalfa *MsRCI2A* significantly enhances salt tolerance in transgenic plants (Fu et al., 2012; Long et al., 2015). Recent studies further demonstrated that alfalfa *MsRCI2B* enhances salt tolerance by regulating Na^+^/K^+^ homeostasis (Qiao et al., 2025; Zhang et al., 2025a). In rice (*Oryza sativa*), an important cereal crop, *RCI2* family members likewise exhibit pronounced responses to abiotic stresses. *OsLti6a* and *OsLti6b* were initially identified as cold-inducible genes (Kim et al., 2007; Morsy et al., 2005). Subsequent studies showed that overexpression of *OsRCI2–5* enhances drought tolerance (Li et al., 2014). In addition, *OsSRLP1* is strongly induced by salt, drought, and cold stresses, and its overexpression promotes free proline accumulation and helps maintain ionic homeostasis, thereby enhancing salt tolerance (Chen et al., 2020). More broadly, RCI2 homologs in diverse plant species have also been reported to respond to various abiotic stresses, including drought and low temperature (Forghani and Aghaie, 2024; Kim et al., 2019, 2022; Senuma et al., 2022; Yang et al., 2025). Collectively, these studies suggest that *RCI2* family members are involved in plant responses to salt, drought, and other abiotic stresses. Further investigation of the *RCI2* family may provide new insights into the molecular mechanisms of abiotic stress tolerance.

Pea is a diploid species (2n = 2x = 14) with seven pairs of chromosomes and a relatively large, repeat-rich genome estimated at approximately 4.45 Gb. Comparative genomic analyses have revealed extensive chromosomal rearrangements, including fusions, fissions, and translocations, during the evolution of pea. Although the pea genome retains signatures of ancient whole-genome duplication events, no recent whole-genome duplication has been detected (Kreplak et al., 2019).

However, although the *RCI2* gene family has been investigated in *Arabidopsis thaliana*, maize, alfalfa, rice, and other plant species, its systematic characterization in pea and its potential roles in responses to salt and osmotic stresses remain largely unexplored. Based on the conserved structural features of RCI2 proteins in other species, we hypothesized that pea *RCI2* genes retain a conserved membrane-associated structure. At the same time, local gene duplication and subsequent regulatory divergence may have contributed to their evolution and distinct responses to salt and osmotic stresses. To test this hypothesis, we systematically identified the *RCI2* gene family in pea and characterized its phylogenetic relationships, chromosomal distribution, gene structures, conserved motifs, protein structural features, and *cis*-acting regulatory elements. We further integrated transcriptome data with salt- and PEG-induced osmotic stress treatments to investigate the expression patterns of *PsRCI2* genes and validated the expression changes of selected candidate genes by quantitative real-time PCR (qRT-PCR). This study provides a systematic characterization of the pea *RCI2* gene family and identifies candidate genes for further investigation of the molecular mechanisms underlying abiotic stress responses in pea.

## 2 Materials and methods

### 2.1 Identification of the *RCI2* gene family

The reference genome sequence and genome annotation file of pea (*Pisum sativum* L.) were downloaded from the National Center for Biotechnology Information (NCBI) under accession number GCF_024323335.1 (Yang et al., 2022). *Arabidopsis* RCI2 protein sequences were first used as queries to perform BLAST searches against the pea genome, with an E-value threshold of 1e-5, to identify preliminary RCI2 candidates. Subsequently, the hidden Markov model (HMM) profile of the conserved PMP3 domain (PF01679) was obtained from the Pfam database (http://pfam.xfam.org/) and used to identify additional candidate RCI2 proteins in TBtools (Chen et al., 2023). The conserved domains of the candidate proteins were further evaluated using the NCBI Conserved Domain Search (CD-Search) tool (https://www.ncbi.nlm.nih.gov/Structure/cdd/wrpsb.cgi) and the InterPro database (https://www.ebi.ac.uk/interpro/). Proteins containing the characteristic PMP3 conserved domain were selected for subsequent analyses. The amino acid number, molecular weight (MW), theoretical isoelectric point (pI), and other physicochemical properties of the identified PsRCI2 proteins were calculated using the Protein Parameter Calc module in TBtools.

### 2.2 Chromosomal localization and phylogenetic analysis

Based on the pea reference genome sequence and corresponding annotation file, the chromosomal locations of *PsRCI2* genes were determined using TBtools and graphically visualized.

Protein sequences of *RCI2* family members from *Arabidopsis thaliana*, alfalfa (*Medicago sativa*), soybean (*Glycine max*), and rice (*Oryza sativa*) were retrieved from the Phytozome database (https://phytozome-next.jgi.doe.gov/). Multiple sequence alignment of RCI2 proteins from pea and the above-mentioned species was performed using ClustalW implemented in MEGA 12 (Kumar et al., 2024). A phylogenetic tree was subsequently constructed using the maximum-likelihood method with 1,000 bootstrap replicates. The resulting phylogenetic tree was subsequently edited and visualized using the Interactive Tree of Life (iTOL) platform (https://itol.embl.de/) (Letunic and Bork, 2024).

### 2.3 Gene structure and motif analysis

The pea genome annotation file was imported into TBtools to visualize the gene structures of *PsRCI2* family members. Conserved motifs in the encoded proteins were identified using the MEME Suite (https://meme-suite.org/meme/tools/meme) (Bailey et al., 2009), with the maximum number of motifs set to four and all other parameters kept at default values. The resulting gene structures and motifs were visualized using TBtools.

### 2.4 Multiple sequence alignment and prediction of protein three-dimensional structures

To investigate sequence conservation within the pea *RCI2* family and across plant species, the six PsRCI2 protein sequences were aligned with the eight *Arabidopsis* RCI2 proteins and two rice RCI2 proteins. Multiple sequence alignment was performed using ClustalW, and the resulting alignment was visualized using GeneDoc. The conserved PMP3 domain was annotated using the InterPro database (https://www.ebi.ac.uk/interpro/) (Blum et al., 2025), and conserved amino acid positions were examined across the aligned sequences.

To compare the predicted structural characteristics of different PsRCI2 members, three-dimensional protein structures were predicted using AlphaFold3 (Jumper et al., 2021) and visualized with PyMOL. Based on transmembrane helix predictions from the TMHMM-2.0 server (https://services.healthtech.dtu.dk/services/TMHMM-2.0/), the predicted transmembrane helices of each PsRCI2 protein were individually color-coded in PyMOL to facilitate comparison of the transmembrane regions and overall predicted conformations among family members.

### 2.5 Collinearity analysis of the PsRCI2 gene family

Based on the pea reference genome sequence and corresponding gene annotation file, intra- and interspecific collinearity analyses of the *RCI2* gene family were performed using TBtools. First, genome-wide collinearity analysis was performed in pea using the MCScanX module implemented in TBtools. Subsequently, *Arabidopsis thaliana*, *Glycine max*, *Medicago truncatula*, and *Oryza sativa* were selected for interspecific collinearity analysis to identify collinear homologs of *PsRCI2* genes and conserved genomic regions. The resulting collinearity maps were visualized using TBtools.

### 2.6 Analysis of *cis*-acting elements in the promoter regions

The 2.0-kb upstream regions relative to the ATG start codon of each *PsRCI2* gene were extracted and submitted to the PlantCARE database (https://bioinformatics.psb.ugent.be/webtools/plantcare/html/) for prediction of *cis*-acting elements (Lescot et al., 2002). The identified *cis*-acting elements were classified according to their functional categories. TBtools was then used to visualize the types, numbers, and relative distributions of *cis*-acting elements within the 2.0-kb upstream regions of the *PsRCI2* genes.

### 2.7 Transcriptome sequencing and data analysis

Uniformly growing Zhongwan 6 pea seedlings at the three-leaf-one-heart stage were selected for salt and PEG-induced osmotic stress treatments. Salt stress was imposed with 150 mM NaCl, whereas osmotic stress was induced with 15% PEG 6000. Aboveground tissues were collected at 3, 6, 12, and 24 h after treatment, while samples collected before treatment (0 h) served as controls. Three biological replicates were included for each treatment. The collected samples were immediately frozen in liquid nitrogen and stored at −80 °C until RNA extraction. Total RNA was then extracted for mRNA enrichment, library preparation, and high-throughput sequencing.

Raw sequencing reads were filtered using fastp v0.23.2, and sequencing quality was assessed using FastQC v0.11.5. The resulting clean reads were aligned to the pea reference genome using HISAT2 v2.2.1. Gene-level read counts were obtained using featureCounts v2.0.1. Raw count data were used for differential expression analysis with DESeq2 v1.34.0, whereas FPKM values were calculated for gene expression profiling and visualization. Differentially expressed genes (DEGs) were identified based on an absolute log_2_ fold change ≥ 1 and an adjusted *P*-value < 0.05.

### 2.8 Quantitative real-time PCR analysis

To analyze the expression profiles of *PsRCI2* genes under abiotic stress conditions, Zhongwan 6 pea seedlings at the three-leaf-one-heart stage were treated with 150 mM NaCl or 15% PEG 6000. Aboveground tissues were collected at 0, 3, 6, 12, and 24 h after treatment for subsequent expression analysis.

Total RNA was extracted using the RNAprep Pure Kit (TIANGEN, Beijing, China), and its concentration and purity were determined. First-strand cDNA was synthesized from 1 μg of total RNA using the FastKing One Step RT-PCR Kit (TIANGEN, Beijing, China). Quantitative real-time PCR (qRT-PCR) was performed using FastFire qPCR SYBR Green Master Mix (TIANGEN, Beijing, China) according to the manufacturer’s instructions. *PsACT* (Knopkiewicz and Wojtaszek, 2019) was used as the reference gene for normalization, and relative expression levels were calculated using the 2^−ΔΔCt^ method. The primers used for qRT-PCR are listed in **Supplementary Table S2**. Data are presented as means ± standard deviation (SD) from three biological replicates (n = 3). For each gene under each treatment, differences among time points were analyzed using one-way analysis of variance (ANOVA), followed by Dunnett’s multiple-comparisons test with the 0 h sample as the control. A *P*-value < 0.05 was considered statistically significant, with *P < 0.05 and **P < 0.01.

### 2.9 Subcellular localization

The subcellular localization of PsRCI2 proteins was investigated by transient expression in *Nicotiana benthamiana*. The full-length coding sequences of *PsRCI2* genes were amplified without the stop codons and ligated into the pRC5-GFP expression vector to generate GFP fusion constructs. For colocalization analysis, *Agrobacterium* strains carrying the PsRCI2-GFP fusion constructs, and the mCherry-tagged ER marker were cultured separately and then mixed prior to agroinfiltration into young *N. benthamiana* leaves. The empty pRC5 vector was used as the negative control. After agroinfiltration, the plants were maintained for 72 h under a 12 h light/12 h dark photoperiod before fluorescence observation. GFP and mCherry fluorescence signals were detected using a Zeiss LSM800 confocal laser scanning microscope (Zeiss, Jena, Germany). The primer sequences used for plasmid construction are listed in **Supplementary Table S2**.

## 3 Results

### 3.1 Identification and characteristics of the pea *RCI2* gene family

A genome-wide search of the pea proteome was performed using the hidden Markov model (HMM) profile of the RCI2 protein family. The candidate RCI2 proteins were subsequently validated by conserved domain analysis. Six RCI2 proteins containing the conserved PMP3 domain were ultimately identified and designated *PsRCI2–1* to *PsRCI2–6* according to their chromosomal positions (**Supplementary Table S1**).

Physicochemical analysis showed that the six pea RCI2 proteins ranged from 54 amino acids in length (PsRCI2–1 to PsRCI2-5) to 77 amino acids (PsRCI2-6), with molecular weights ranging from 5,903.37 Da (PsRCI2-2) to 8,867.66 Da (PsRCI2-6). Their theoretical isoelectric points (pI) ranged from 4.00 in PsRCI2–1 to 7.69 in PsRCI2-2. The instability index ranged from 32.84 in PsRCI2–2 to 53.10 in PsRCI2-6. Among the six proteins, PsRCI2–1 and PsRCI2–2 had instability indices below 40 and were therefore predicted to be stable, whereas the remaining members were predicted to be unstable. The aliphatic index ranged from 126.62 in PsRCI2–6 to 164.07 in PsRCI2-1. The grand average of hydropathicity (GRAVY) values of all six proteins were greater than 0, indicating that all six PsRCI2 proteins are predominantly hydrophobic.

### 3.2 Chromosomal localization and phylogenetic analysis

The six identified *PsRCI2* genes were distributed across three pea chromosomes (**Figure 1A**). *PsRCI2–1* was located on chromosome 1, whereas *PsRCI2–2* to *PsRCI2–5* were clustered on chromosome 3, forming a gene cluster. *PsRCI2–6* was located on chromosome 4.

**Figure 1:**
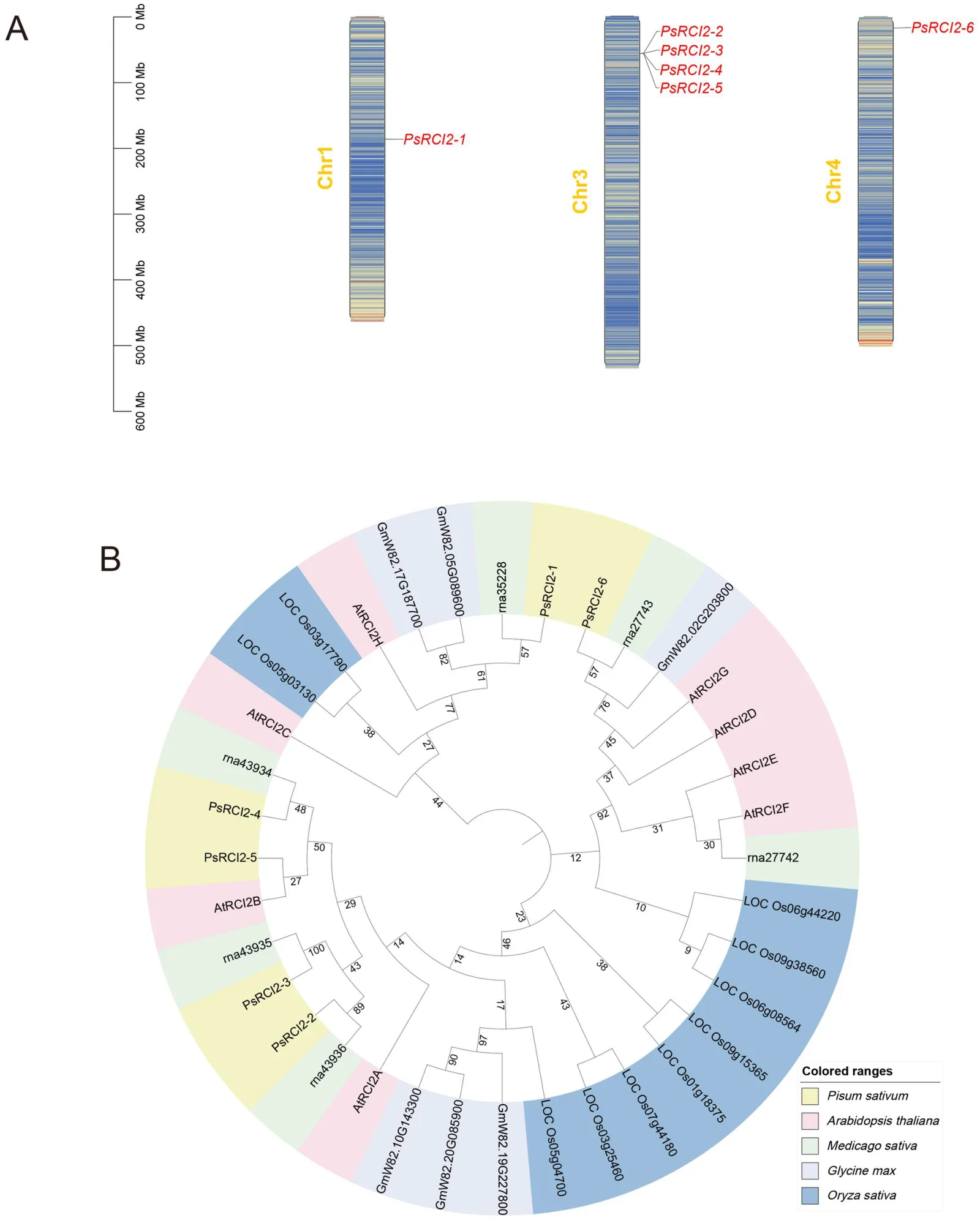
Chromosomal distribution and phylogenetic analysis of the *PsRCI2* gene family. **(A)** Physical mapping of *PsRCI2* genes on pea chromosomes. Chromosomal gene density was calculated using a 1,000-kb sliding window, with the color gradient from blue to red denoting low to high density. **(B)** Phylogenetic tree of RCI2 proteins from *Pisum sativum*, *Arabidopsis thaliana*, *Medicago sativa*, *Glycine max*, *Oryza sativa*. The tree was constructed based on protein sequence alignments. Numbers at the nodes represent bootstrap support values from 1,000 replicates. In the phylogenetic tree, proteins belonging to the same species are uniformly color-coded.

To investigate the evolutionary relationships of the pea *RCI2* family, a phylogenetic tree was constructed using RCI2 protein sequences from pea, *Arabidopsis thaliana*, alfalfa, soybean, and rice (**Figure 1B**). Phylogenetic analysis showed that RCI2 proteins from the five species were distributed among several distinct evolutionary clades. The six PsRCI2 proteins were distributed among distinct clades and clustered with homologous RCI2 proteins from other plant species. Notably, PsRCI2–2 to PsRCI2–5 clustered closely within the same major clade, consistent with their physical clustering on chromosome 3. In contrast, PsRCI2–1 and PsRCI2–6 were located in distinct clades and exhibited closer evolutionary relationships with RCI2 homologs from other legume or dicot species. Overall, the phylogenetic analysis revealed distinct evolutionary relationships among PsRCI2 proteins and their homologs across plant species.

### 3.3 Conserved motifs and gene structures analysis

The conserved motif composition and gene structures of the six *PsRCI2* members were analyzed to assess their structural conservation. Four conserved motifs were identified (**Figure 2A**, **Supplementary Figure S1**). PsRCI2–2 to PsRCI2–5 shared highly similar motif compositions and arrangements, predominantly consisting of Motifs 2, 3, 1, and 4. PsRCI2–1 and PsRCI2–6 exhibited a similar motif pattern but lacked the terminal Motif 4. Consistent with the motif composition, *PsRCI2–2* to *PsRCI2–5* exhibited highly similar gene structures (**Figure 2B**). In contrast, *PsRCI2–1* and *PsRCI2–6* differed from these four members in gene length and structural organization, whereas *PsRCI2–3* was characterized by a relatively long 3′ UTR. Overall, the conserved motif arrangements and similar exon–intron organizations observed among most *PsRCI2* members indicate a high degree of structural conservation within the pea *RCI2* family.

**Figure 2:**
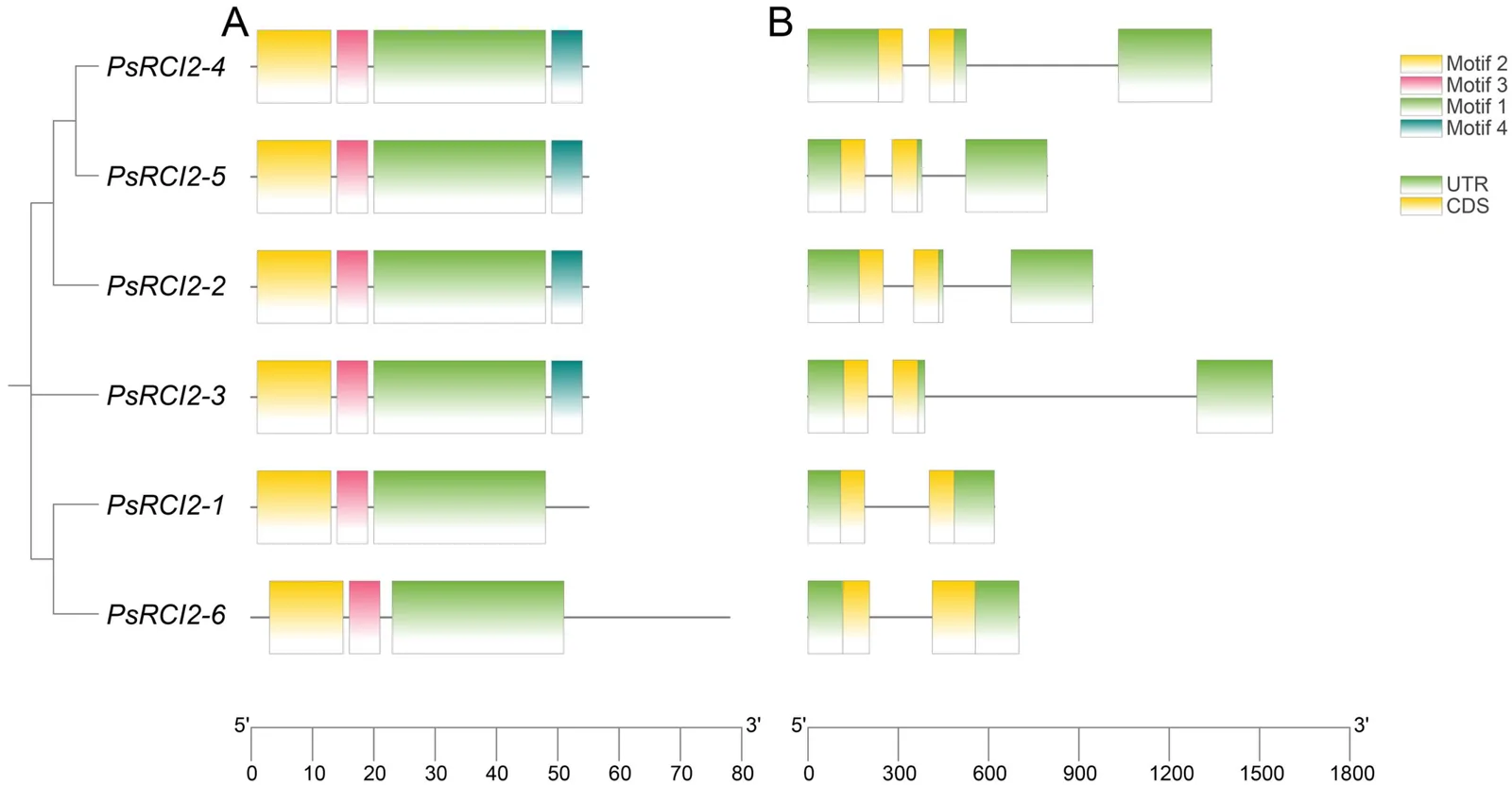
Structural analysis of the *PsRCI2* gene family. **(A)** Conserved motif patterns of PsRCI2 proteins. Four motifs are shown in different colors. **(B)** Exon–intron structures of *PsRCI2* genes, green boxes indicate UTRs, yellow boxes indicate CDSs, and thin gray lines represent introns.

### 3.4 Conserved domains and predicted structures of PsRCI2 proteins

Multiple sequence alignment of the six PsRCI2 proteins with representative RCI2 homologs from *Arabidopsis* and rice revealed conserved sequence features within the PMP3 domain (**Figure 3A**). The cross-species comparison also enabled the conserved regions of the pea proteins to be evaluated in an evolutionary context. PsRCI2–1 to PsRCI2–5 exhibited highly similar protein sequences, with each protein comprising 54 amino acids and sharing highly similar transmembrane regions. In contrast, PsRCI2–6 was 77 amino acids long and contained an additional C-terminal extension, with only one transmembrane domain predicted, distinguishing it from the other PsRCI2 members.

**Figure 3:**
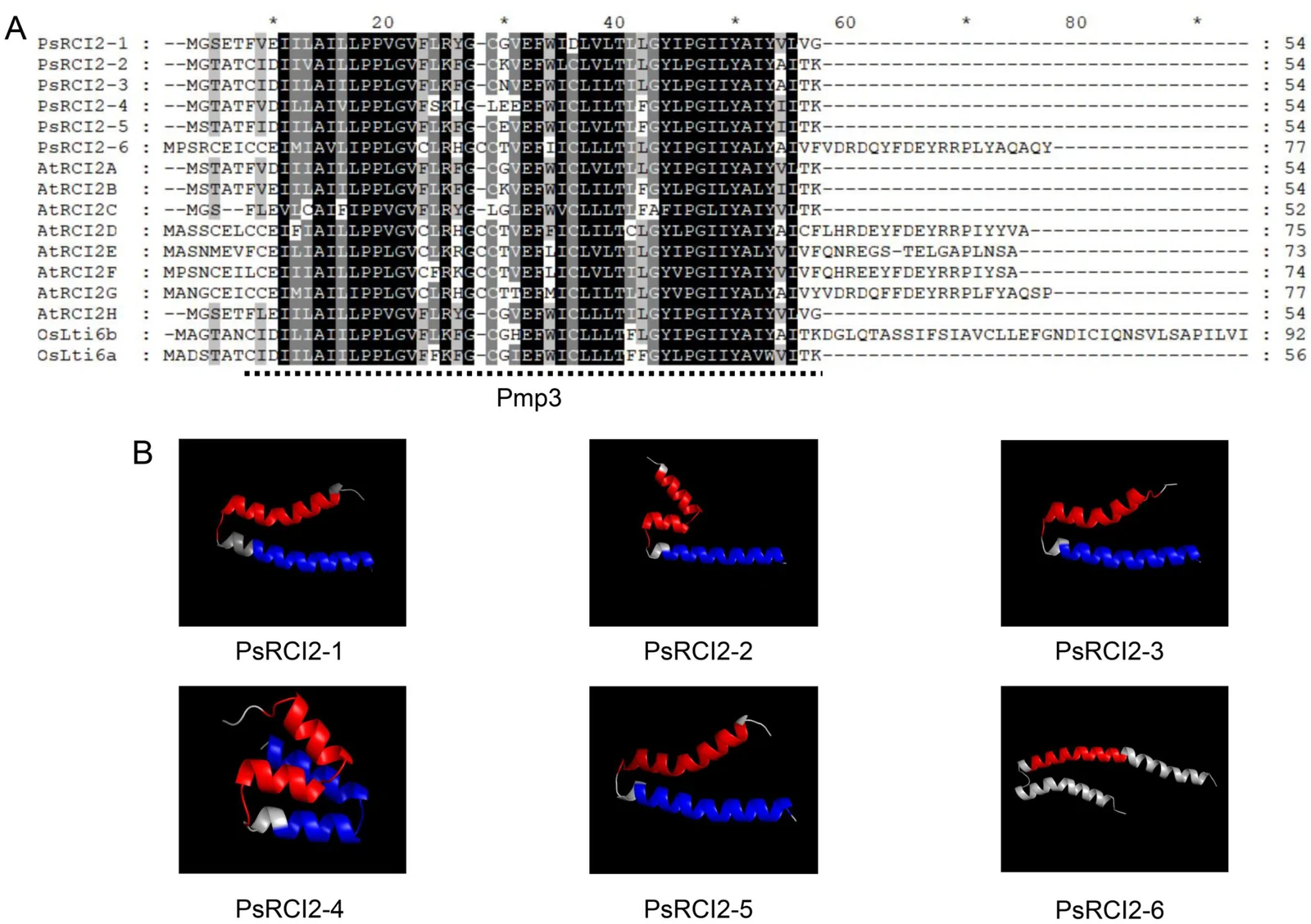
Sequence conservation and predicted transmembrane structures of RCI2 proteins. **(A)** Multiple sequence alignment of six pea RCI2 proteins, eight *Arabidopsis* RCI2 proteins, and two rice RCI2 proteins. The PMP3 domain is indicated by a black dashed line. **(B)** Predicted three-dimensional structures of PsRCI2–1 to PsRCI2-6. Predicted transmembrane helices are highlighted in red and blue, where applicable, whereas non-transmembrane regions are shown in gray.

Three-dimensional structure prediction further showed that PsRCI2–1 to PsRCI2–5 predominantly adopted similar α-helical conformations, consistent with the high conservation of their transmembrane regions (**Figure 3B**). The structure of PsRCI2–4 showed some differences from those of the other members. PsRCI2–6 was more distinct, with an extended C-terminal region and a different number of predicted transmembrane domains. Overall, the *PsRCI2* family exhibited substantial conservation in its core structural regions, whereas PsRCI2–6 showed greater divergence in predicted structure, protein length, and transmembrane architecture.

### 3.5 Intraspecific and interspecific collinearity analysis

To investigate the duplication history and evolutionary patterns of the *PsRCI2* gene family within the pea genome, genome-wide intraspecific collinearity analysis was first performed (**Figure 4A**). The results revealed extensive collinear blocks among pea chromosomes, suggesting a complex history of genome duplication and rearrangement in pea. However, no clear collinear gene pairs involving *PsRCI2* genes were identified within these regions. Because segmental duplication is generally inferred from homologous genes retained in corresponding positions within duplicated collinear blocks, this finding provides no direct evidence that detectable segmental duplication made a major contribution to the current *PsRCI2* gene family.

**Figure 4:**
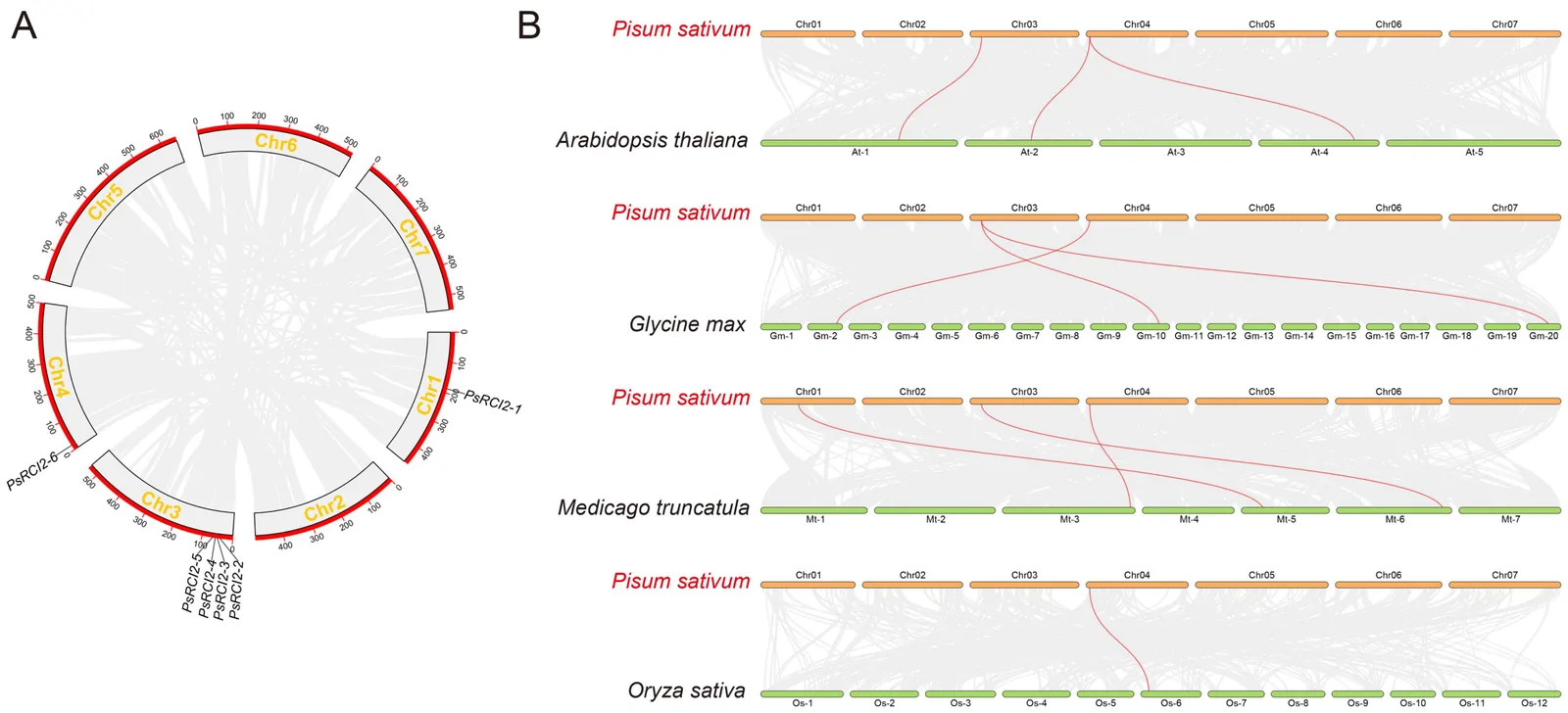
Synteny analysis of the *PsRCI2* gene family. **(A)** Intraspecific synteny analysis of *PsRCI2* genes in the pea genome. Gray lines represent syntenic blocks across the genome. **(B)** Comparative synteny analysis of *RCI2* genes between *P. sativum* and *A. thaliana*, *G. max*, *M. truncatula*, and *O. sativa*, respectively. Gray lines represent collinear genomic blocks between pea and the comparison species, whereas red lines indicate orthologous gene pairs.

To further investigate the evolutionary relationships of the *RCI2* family across plant species, interspecific collinearity analysis was performed (**Figure 4B**). Three collinear *RCI2* gene pairs were identified between pea and *Arabidopsis thaliana*, soybean, and *Medicago truncatula*, whereas only one collinear *RCI2* gene pair was identified between pea and rice.

### 3.6 Analysis of *cis*-acting elements in the promoter regions

To investigate the potential transcriptional regulation of *PsRCI2* genes under abiotic stress, the 2.0-kb upstream regions relative to the ATG start codon of the six *PsRCI2* genes were analyzed for *cis*-acting regulatory elements (**Figure 5**). Numerous *cis*-acting elements associated with phytohormone responses, abiotic stress responses, and plant growth and development were identified in all six *PsRCI2* promoters, suggesting that these genes may be subject to complex transcriptional regulation.

**Figure 5:**
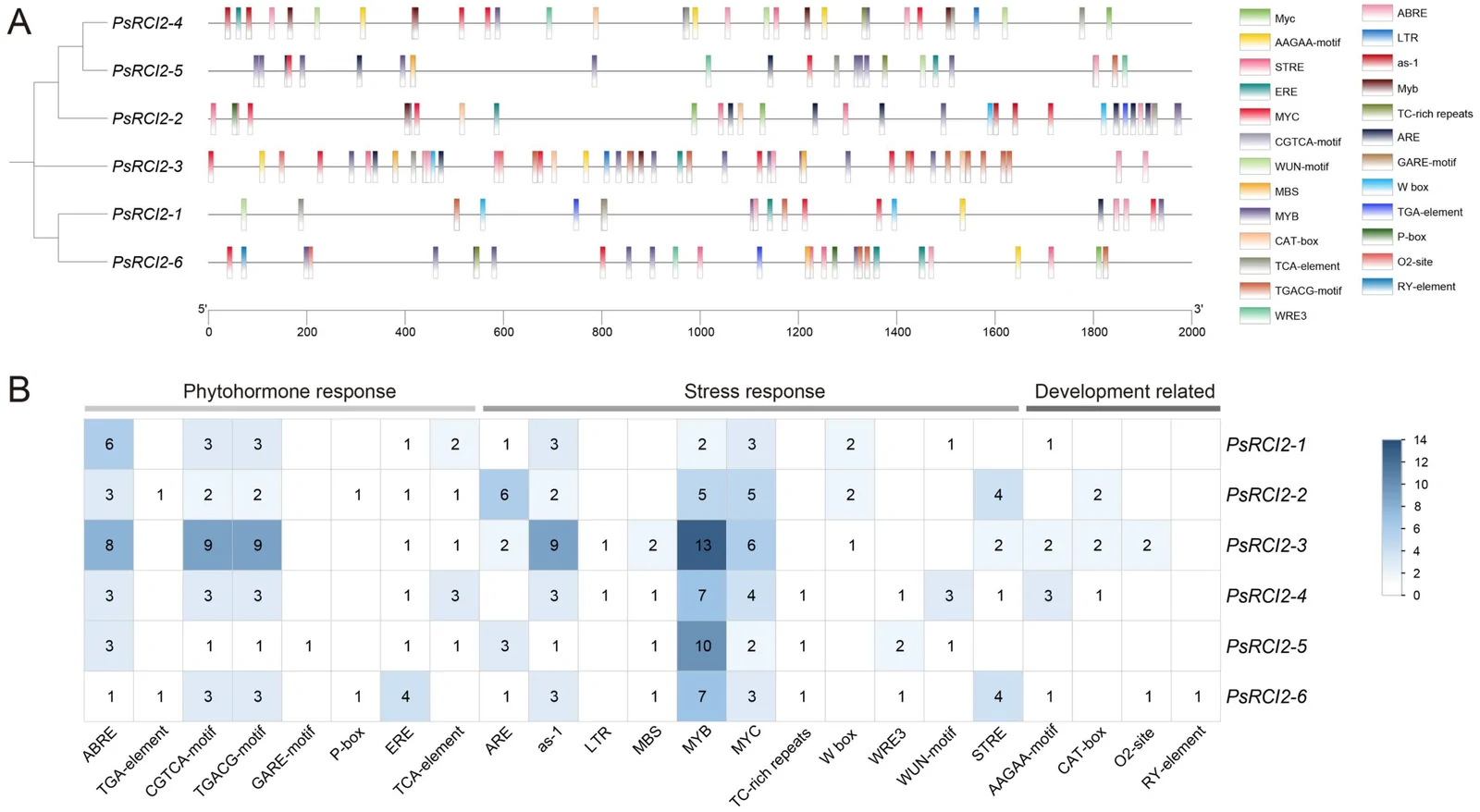
Distribution of *cis*-acting elements in the promoter regions of the *PsRCI2* gene family. **(A)** *C*is-acting elements predicted within the 2.0-kb upstream regions of the *PsRCI2* genes relative to the ATG start codons. All displayed elements are located on the sense strand. **(B)** Heatmap values indicate the copy number of each corresponding *cis*-acting element.

Hormone-responsive elements included ABRE, TGA-element, CGTCA-motif, TGACG-motif, GARE-motif, P-box, ERE, and TCA-element. Stress-responsive elements included ARE, as-1, LTR, MBS, MYB, MYC, W-box, WRE3, and STRE. In addition, several cis-acting elements associated with plant growth and development were identified, including WUN-motif, AAGAA-motif, CAT-box, O2-site, and RY-element.

The abundance and distribution of these elements varied considerably among the six *PsRCI2* promoters. Notably, the *PsRCI2–3* promoter was enriched in both hormone- and stress-responsive elements, including eight ABRE elements, nine CGTCA-motifs, nine TGACG-motifs, and 13 MYB elements. MYC elements were detected in all six promoters, with copy numbers ranging from 2 to 6, whereas other regulatory elements showed variable abundance among family members.

Overall, the distinct composition and distribution of *cis*-acting elements among *PsRCI2* promoters, particularly the enrichment of hormone- and stress-responsive elements in the *PsRCI2–3* promoter, suggest that *PsRCI2* genes may be differentially regulated under abiotic stress conditions. These findings provide a basis for further investigating the transcriptional regulation of *PsRCI2* genes and their potential roles in abiotic stress responses.

### 3.7 Expression of *PsRCI2* genes under salt and osmotic stress

To investigate the responses of the *PsRCI2* gene family to salt and osmotic stresses, the expression patterns of the six *PsRCI2* genes at different time points were analyzed based on transcriptome sequencing data. A hierarchical clustering heatmap was generated based on normalized expression values (**Figure 6**). The results showed that *PsRCI2* family members exhibited distinct transcriptional responses to salt and PEG treatments.

**Figure 6:**
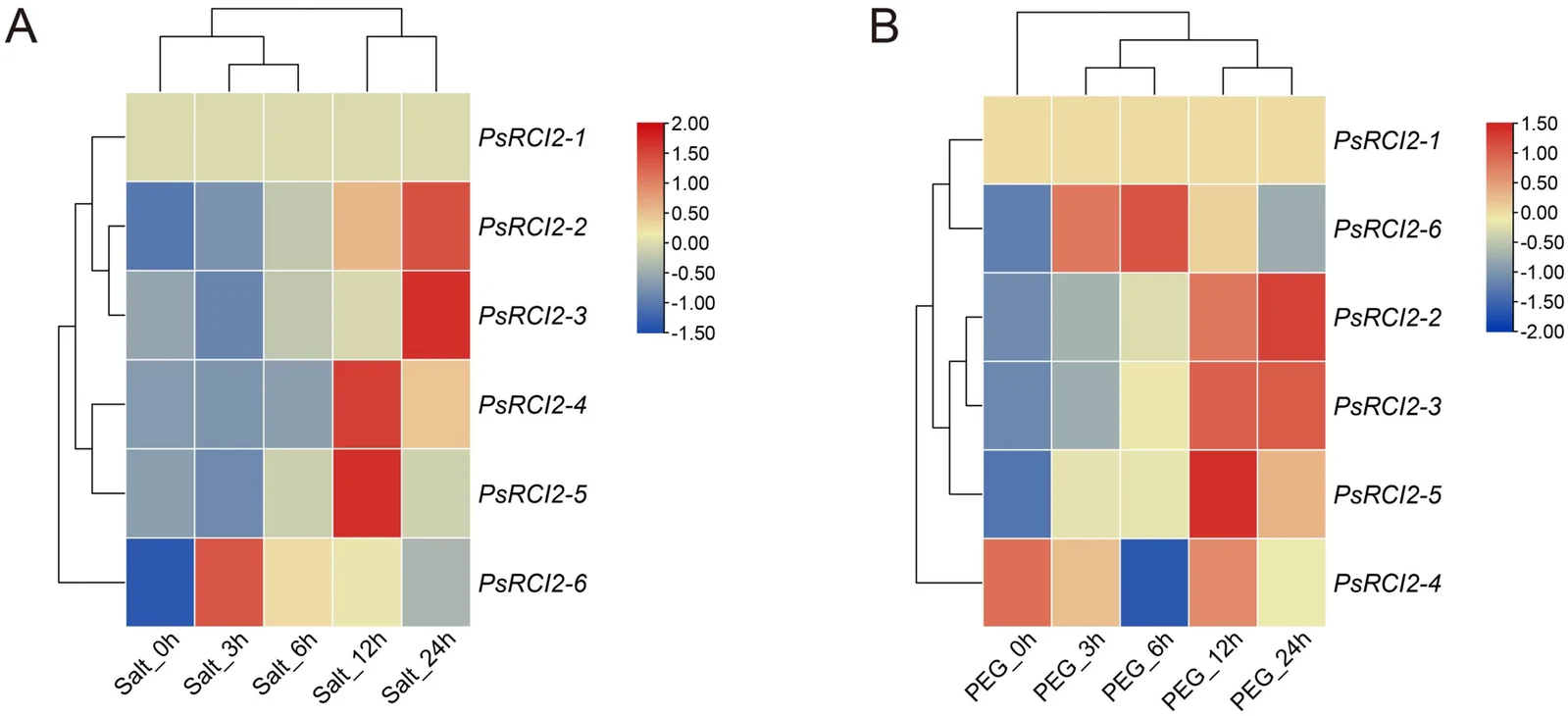
Transcriptome-based expression profiles of *PsRCI2* genes in response to salt and osmotic stresses. **(A)** Expression patterns of the six *PsRCI2* genes under salt treatment at 0, 3, 6, 12, and 24 h. **(B)** Expression patterns of the six *PsRCI2* genes under PEG treatment at the corresponding time points. Red and blue indicate relatively high and low normalized transcript abundance, respectively. The heatmap illustrates relative expression patterns across treatment time points.

Under salt stress, *PsRCI2–2* to *PsRCI2–5* generally showed increasing transcript abundance, whereas *PsRCI2–6* showed a strong early induction and *PsRCI2–1* remained relatively stable. Under PEG treatment, *PsRCI2-2*, *PsRCI2-3*, and *PsRCI2–5* showed increasing transcript abundance, while *PsRCI2–6* also responded rapidly during the early treatment stage. *PsRCI2–1* and *PsRCI2–4* showed no obvious response to PEG treatment.

Comparison of the two treatments revealed both shared and treatment-dependent expression patterns. *PsRCI2-2*, *PsRCI2-3*, and *PsRCI2–5* responded to both salt and PEG treatments, suggesting that these genes may participate in responses shared between salt and osmotic stress. *PsRCI2–6* showed an early response under both conditions, whereas *PsRCI2–4* appeared more responsive to salt than to PEG treatment. By comparison, *PsRCI2–1* exhibited relatively limited changes under either condition. Overall, the six family members differed in their apparent response timing and relative expression changes, indicating that the *PsRCI2* family exhibits both shared and treatment-dependent transcriptional responses to abiotic stress.

### 3.8 Validation of transcriptome data by qRT-PCR

To validate the transcriptome sequencing results, *PsRCI2-2*, *PsRCI2-3*, and *PsRCI2–5* were selected for qRT-PCR analysis (**Figure 7**). The results showed that all three genes were markedly induced by both salt and PEG-induced osmotic stresses, although their temporal expression patterns differed.

**Figure 7:**
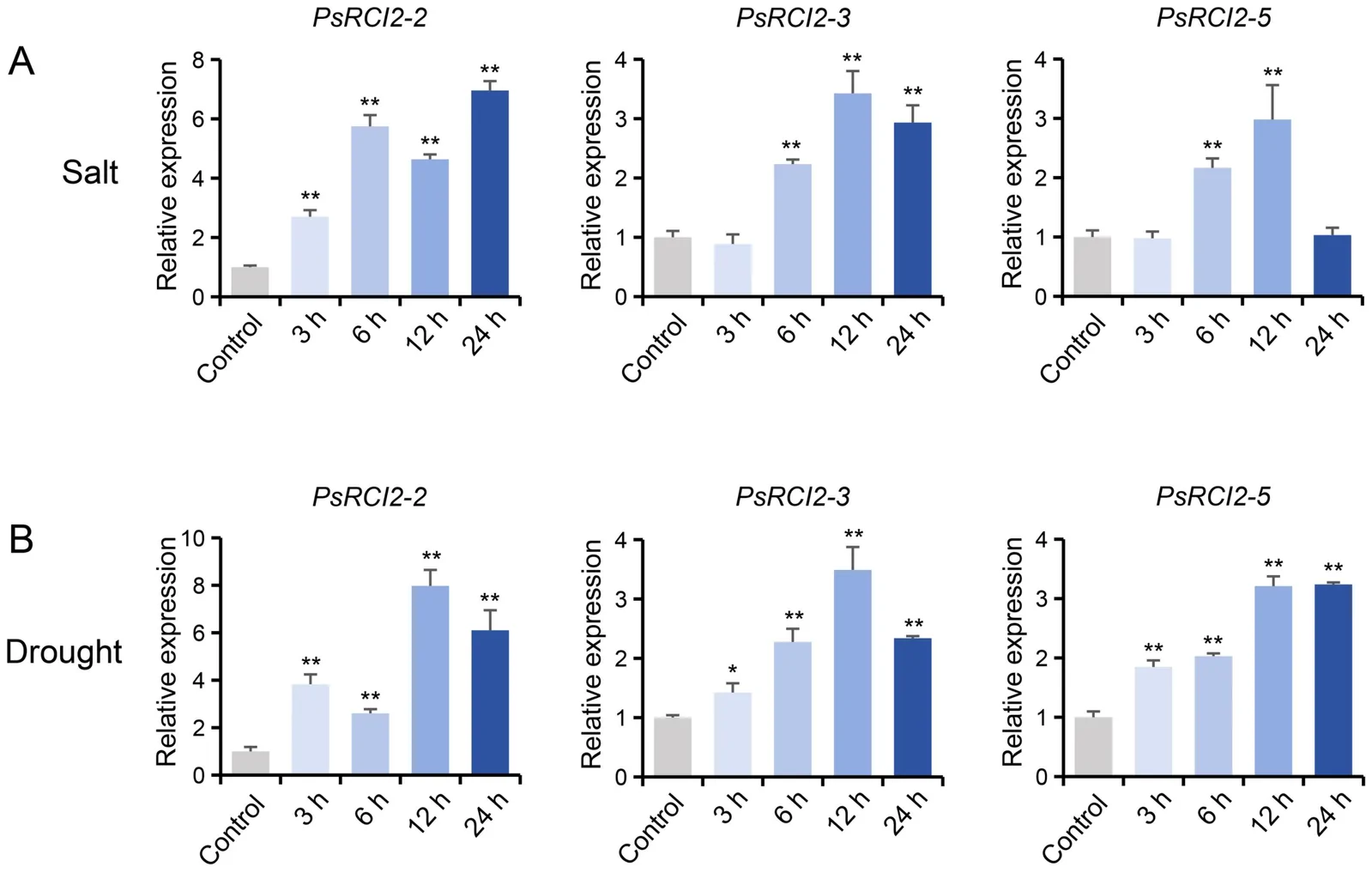
Relative expression levels of *PsRCI2-2*, *PsRCI2-3*, and *PsRCI2–5* under salt **(A)** and PEG-induced osmotic **(B)** stress at 0, 3, 6, 12, and 24 h. Data are presented as means ± SD from three biological replicates (n = 3). For each gene under each treatment, statistical significance was evaluated using one-way ANOVA followed by Dunnett’s multiple-comparisons test, with the corresponding 0 h sample used as the control. Asterisks indicate significant differences compared with the 0 h control (*P < 0.05; **P < 0.01).

Under salt stress, *PsRCI2–2* expression increased continuously and reached its highest level at 24 h. *PsRCI2–3* and *PsRCI2–5* both exhibited relatively high expression levels at 12 h. *PsRCI2–3* remained highly expressed at 24 h, whereas *PsRCI2–5* expression decreased markedly at 24 h. Under PEG treatment, *PsRCI2–2* and *PsRCI2–3* both reached peak expression levels at 12 h. In contrast, *PsRCI2–5* expression increased continuously and remained relatively high from 12 to 24 h.

The qRT-PCR results were generally consistent with the transcriptome data in terms of the direction of expression changes, although the magnitude of changes differed between the two approaches. Given their responsive patterns to both salt and osmotic stresses, *PsRCI2-2*, *PsRCI2-3*, and *PsRCI2–5* were considered promising candidate genes for further functional characterization.

### 3.9 Subcellular localization analysis

To determine the subcellular localization of representative PsRCI2 proteins, *PsRCI2-2*, *PsRCI2-3*, and *PsRCI2–5* were selected for transient expression analysis based on their pronounced transcriptional responses to salt and osmotic stresses. The coding sequences of *PsRCI2-2*, *PsRCI2-3*, and *PsRCI2–5* were fused in frame with GFP and transiently expressed in leaf epidermal cells of *N. benthamiana*. PsRCI2-2–GFP, PsRCI2-3–GFP, and PsRCI2-5–GFP showed fluorescence patterns that overlapped with the ER marker (**Figure 8**). These observations indicate that the three PsRCI2-GFP fusion proteins exhibit ER-associated fluorescence in *N. benthamiana* leaf epidermal cells under the conditions examined. However, the possibility that GFP fusion affects their subcellular distribution cannot be excluded.

**Figure 8:**
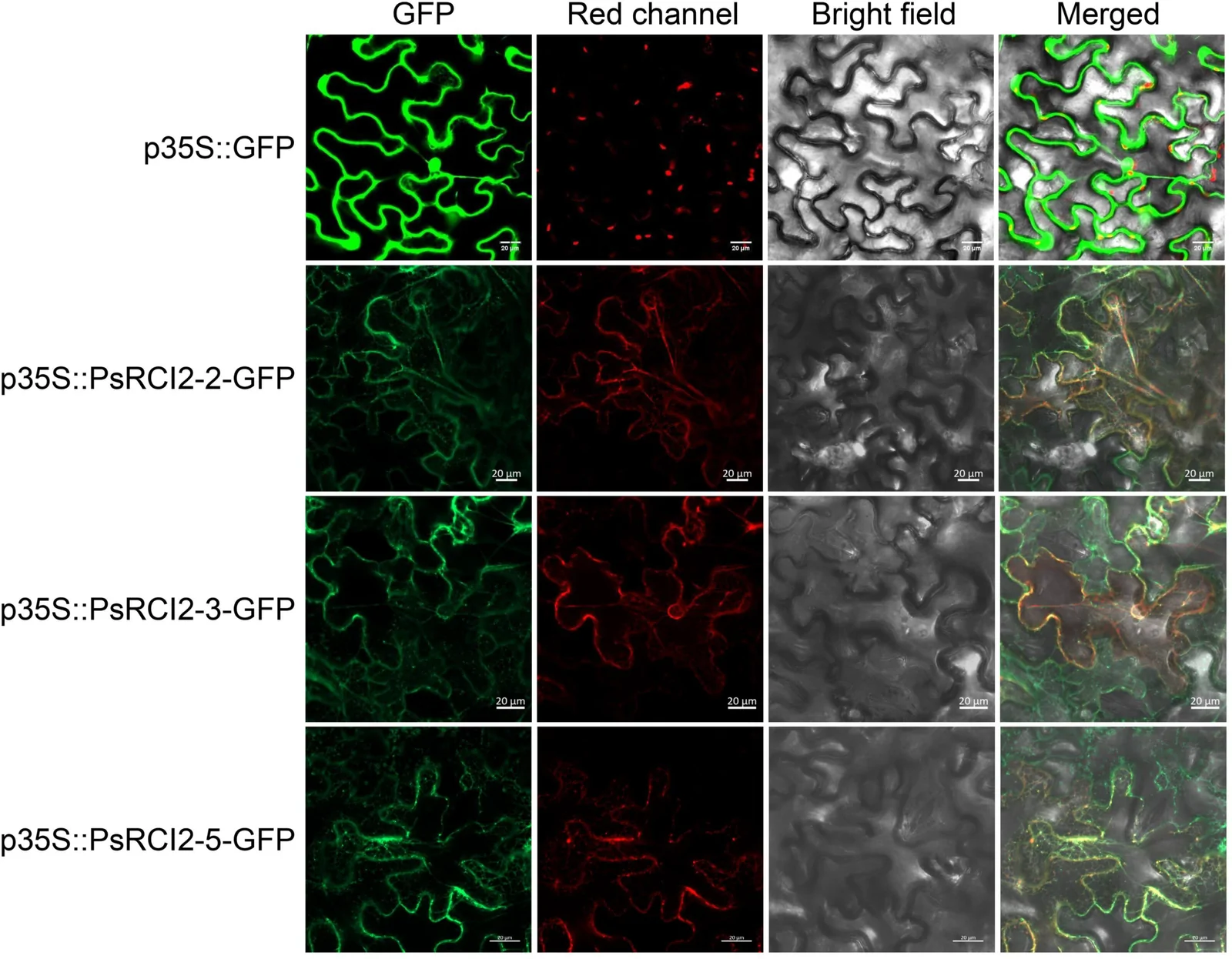
Subcellular fluorescence patterns of GFP-tagged PsRCI2 proteins in *N. benthamiana* leaves. GFP fluorescence, ER marker fluorescence, bright field images, and merged images are shown from left to right. In the p35S::GFP control, the red channel signal represents chlorophyll autofluorescence, whereas in leaves expressing PsRCI2-GFP fusion proteins, the red fluorescence corresponds to the ER marker signal. Scale bar, 20 μm.

## 4 Discussion

*RCI2* genes show dynamic expression patterns, whereas their encoded proteins exhibit diverse subcellular distributions and trafficking behaviors (Kim et al., 2021; Martinière et al., 2019; Medina et al., 2007). However, the evolutionary characteristics and stress-related functions of the *RCI2* family in pea have not been well characterized. In this study, six *PsRCI2* genes were identified and analyzed using phylogenetic, structural, promoter, transcriptomic, qRT-PCR, and subcellular localization approaches. Results showed that the PsRCI2 proteins share a conserved overall architecture, whereas their promoter structures and expression patterns differ considerably among family members. In particular, several *PsRCI2* genes responded strongly to salt and osmotic stress, although the magnitude and timing of their responses varied. These findings indicate that the pea *RCI2* family has retained conserved structural features during evolution while acquiring distinct regulatory characteristics, which may contribute to the functional diversification of individual *PsRCI2* genes under abiotic stress.

The six *PsRCI2* genes are unevenly distributed across the pea genome, with *PsRCI2–2* to *PsRCI2–5* located in a relatively compact region on chromosome 3, while *PsRCI2–1* and *PsRCI2–6* are located on chromosomes 1 and 4, respectively. The close phylogenetic relationship among *PsRCI2–2* to *PsRCI2–5* is consistent with their physical proximity and suggests that local duplication may have contributed to their organization. However, no clear collinear *PsRCI2* pairs were identified within the pea genome, providing limited evidence for a major contribution of detectable segmental duplication to the current family composition. This inference should be interpreted cautiously because ancient duplication events may become difficult to detect following gene loss, sequence divergence, or chromosomal rearrangements. The clustered *PsRCI2* genes also showed differences in their transcriptional responses to salt and osmotic stresses, suggesting that genes with a common evolutionary origin may have subsequently acquired distinct regulatory patterns. Such divergence could contribute to functional differentiation among closely related family members.

The structural features of PsRCI2 proteins revealed a high degree of conservation among most family members. PsRCI2–1 to PsRCI2–5 showed similar amino acid sequences and predicted transmembrane conformations, consistent with the conserved structural features reported for RCI2 proteins (Kim et al., 2021). The conserved hydrophobic regions may contribute to membrane association and structural integrity. In contrast, PsRCI2–6 differed from the other members, with a longer C-terminal region and only one predicted transmembrane helix. This structural difference, together with its distinct stress-responsive expression pattern, suggests that PsRCI2–6 may have undergone some degree of functional differentiation from the other family members. However, the three-dimensional structures and transmembrane topology analyzed here were predicted computationally and have not been experimentally validated. Therefore, the structural differences observed for PsRCI2–6 should be regarded as potential features associated with functional divergence rather than direct evidence of distinct biochemical functions.

Promoter analysis revealed substantial differences in the *cis*-regulatory composition of the *PsRCI2* genes, which may contribute to their distinct transcriptional responses to stress. Multiple hormone- and stress-related *cis*-elements, including ABRE, MYB, MYC, and W-box elements, were identified in the promoters, suggesting that *PsRCI2* expression may be regulated by diverse stress- and hormone-associated signaling pathways (Sheshadri et al., 2016; Zou et al., 2011). *PsRCI2–3* contained a relatively high number of several stress- and hormone-responsive elements and also showed a strong and sustained response to both salt and PEG treatments. These observations suggest that differences in promoter composition may contribute to the divergent expression patterns of *PsRCI2* members.

To further explore the potential regulatory mechanisms underlying these expression patterns, the transcriptome profiles were examined for stress-responsive transcription factors. Among the candidate TFs, *LOC127093549* and *LOC127091880*, which belong to the MYB gene family, showed increased expression under salt and PEG treatments, coinciding with the expression responses of *PsRCI2-2/3/5*. The presence of MYB *cis*-element in the promoter of *PsRCI2-2/3/5* provides a possible link between these candidate TFs and the observed transcriptional response. This association suggests that *LOC127093549* and *LOC127091880* may be involved in the regulation of *PsRCI2-2/3/5* under the corresponding stress condition. Nevertheless, this relationship remains hypothetical because co-expression and predicted *cis*-elements do not establish direct TF binding or transcriptional regulation. Further experimental validation will be required to determine whether these candidate TFs directly regulate the expression of the corresponding *PsRCI2* genes.

A central finding of this study is that *PsRCI2-2*, *PsRCI2-3*, and *PsRCI2–5* showed clear transcriptional responses to both salt and PEG treatments, suggesting that these genes may be involved in the pea response to osmotic stress. Their responses to both treatments are noteworthy because, although salt stress involves both osmotic and ionic components, PEG treatment primarily imposes an osmotic constraint (Zhu, 2016). Thus, their shared response may reflect involvement in osmotic stress responses or related stress-adaptation processes rather than responses restricted to ionic stress. This observation is in line with previous studies showing that RCI2 proteins are associated with membrane and ionic homeostasis and contribute to salt or drought responses in *Arabidopsis*, maize, alfalfa, and rice (Lei et al., 2023; Medina et al., 2005; Chen et al., 2020; Qiao et al., 2025). Despite their close sequence similarity, *PsRCI2-2*, *PsRCI2-3*, and *PsRCI2–5* differed in the timing and magnitude of transcriptional induction, indicating that these genes are not regulated identically under stress. Such differences may allow closely related *PsRCI2* members to respond at different stages of stress treatment or under different regulatory contexts. The transcriptome and qRT-PCR results showed generally consistent expression trends for the selected genes, although the magnitude of changes differed between the two approaches. This difference may reflect the distinct normalization and quantification methods used for transcriptome sequencing and qRT-PCR. However, their stress-induced expression alone is insufficient to establish a direct role in stress tolerance, and functional validation will be required to determine their specific contributions.

The subcellular localization results provide further insight into the potential functions of PsRCI2 proteins. PsRCI2-2–GFP, PsRCI2-3–GFP, and PsRCI2-5–GFP showed fluorescence patterns that largely overlapped with the ER marker in *N. benthamiana*. This observation should be interpreted cautiously because RCI2 proteins are generally associated with the plasma membrane, although intracellular localization has also been reported for some family members (Kim et al., 2021; Medina et al., 2007). Given the small size of the PsRCI2 proteins, fusion to the relatively large GFP tag may affect their folding, trafficking, or subcellular distribution. Therefore, the observed fluorescence supports an ER-associated pattern of the GFP fusion proteins but does not conclusively establish the native localization of the untagged proteins or exclude possible plasma membrane localization. Further validation using an independent plasma membrane marker, such as PIP2;1, or a smaller epitope tag would help distinguish genuine ER association from potential tag-dependent effects. Until such validation is available, the localization results should be considered preliminary.

More broadly, although transcriptome analysis, qRT-PCR, and subcellular localization provide evidence of stress-responsive expression and membrane-associated distribution, the present study does not establish the direct contribution of individual *PsRCI2* genes to salt or osmotic stress tolerance in pea. The identified genes should therefore be regarded as candidates for further functional characterization. Future studies using yeast complementation and genetic manipulation in pea or a suitable model plant, together with measurements of ion accumulation, membrane potential, and stress-related physiological traits, will be needed to determine whether these proteins directly contribute to stress tolerance and to clarify their underlying mechanisms.

Overall, this study identifies the *PsRCI2* family as a structurally conserved yet transcriptionally diverse group in pea. The clustering of *PsRCI2–2* to *PsRCI2–5* on chromosome 3, together with the absence of clear intragenomic collinear pairs, suggests that local duplication may have contributed to the organization of this family, although a major contribution from segmental duplication was not supported by the present analysis. The structural divergence of *PsRCI2–6* and the differences in stress-responsive expression among family members further highlight the diversity of the pea RCI2 family. In particular, *PsRCI2-2*, *PsRCI2-3*, and *PsRCI2–5* emerged as promising candidates for further investigation based on their responses to salt and PEG treatments. These findings provide a foundation for future studies aimed at elucidating the molecular mechanisms underlying RCI2-mediated abiotic stress responses in pea.

## Data Availability

The data presented in the study are deposited in the NCBI Sequence Read Archive (SRA) under BioProject accession number PRJNA1548852.
