# Supplementary material for: Genome-wide characterization of the RCI2 gene family reveals conserved protein features and divergent responses to salt and osmotic stress in pea
Source: Front Plant Sci. 2026 Oct 29;17:2003888. doi: 10.3389/fpls.2026.2003888 (PMC13754750; doi:10.3389/fpls.2026.2003888)
Supplement: Supplementary file 1 [file SupplementaryFile1.docx]

**Supplementary Figure S1.** Conserved motifs of the *PsRCI2* gene family.

**Supplementary Table S1.** Identification information of PsRCI2 proteins

| **ID** | **Number of Amino Acid (AA)** | **Molecular Weight (Da)** | **Theoretical pI** | **Instability Index** | **Aliphatic Index** | **Grand Average of Hydropathicity** |
| --- | --- | --- | --- | --- | --- | --- |
| PsRCI2-1 | 54 | 5931.21 | 4 | 35.23 | 164.07 | 1.511 |
| PsRCI2-2 | 54 | 5903.37 | 7.69 | 32.84 | 158.89 | 1.519 |
| PsRCI2-3 | 54 | 5917.35 | 5.82 | 40.83 | 162.59 | 1.576 |
| PsRCI2-4 | 54 | 6004.35 | 4.41 | 42.88 | 158.89 | 1.378 |
| PsRCI2-5 | 54 | 6068.5 | 4.68 | 44.87 | 158.89 | 1.561 |
| PsRCI2-6 | 77 | 8867.66 | 5.05 | 53.1 | 126.62 | 0.803 |

**Supplementary Table S2.** Primers used in this study

| Purpose | Primer name | Primer sequence (5′ – 3′) |
| --- | --- | --- |
| Subcellular localization | *PsRCI2-2*-pRC5-CF | AttaacagtctagaggtaccATGGGTACAGCTACCTGCAT |
|  | *PsRCI2-2*-pRC5-CR | GaaccaccgccaccggatccTTTAGTGATAGCATAGATGG |
|  | *PsRCI2-3*-pRC5-CF | AttaacagtctagaggtaccATGGGCACTGCTACCTGCAT |
|  | *PsRCI2-3*-pRC5-CR | GaaccaccgccaccggatccCTTAGTGATAGCATAAATTG |
|  | *PsRCI2-5*-pRC5-CF | AttaacagtctagaggtaccATGAGTACAGCTACCTTCAT |
|  | *PsRCI2-5*-pRC5-CR | GaaccaccgccaccggatccCTTAGTGATAATGTAAATAG |
| qRT-PCR | *PsACT*-qPCR-F | CAATGGAACTGGAATGGT |
|  | *PsACT*-qPCR-R | TTAGACTGTGCCTCATCA |
|  | *PsRCI2-2*-qPCR-F | TACCTGCATCGATATCATCG |
|  | *PsRCI2-2*-qPCR-R | AGAGCCAGAACTCAACTTTG |
|  | *PsRCI2-3*-qPCR-F | CTACCTGCATCGATATTATCC |
|  | *PsRCI2-3*-qPCR-R | TAGCCCAAAATGGTGAGAATC |
|  | *PsRCI2-5*-qPCR-F | TGAGTACAGCTACCTTCATTG |
|  | *PsRCI2-5*-qPCR-R | GATCCAAAACTCCACTTCAC |
